# Influence of Mechanical and Mineralogical Activation of Biomass Fly Ash on the Compressive Strength Development of Cement Mortars

**DOI:** 10.3390/ma14216654

**Published:** 2021-11-04

**Authors:** Jakub Popławski, Małgorzata Lelusz

**Affiliations:** Department of Construction and Road Engineering, Faculty of Civil Engineering and Environmental Sciences, Bialystok University of Technology, 15-351 Bialystok, Poland; m.lelusz@pb.edu.pl

**Keywords:** fly ash, cement, activation, biomass

## Abstract

Biomass combustion is a significant new source of green energy in the European Union. The adequate utilization of byproducts created during that process is a growing challenge for the energy industry. Biomass fly ash could be used in cement composite production after appropriate activation of that material. This study had been conducted to assess the usefulness of mechanical and physical activation methods (grinding and sieving), as well as activation through the addition of active silica in the form of silica fume, as potential methods with which to activate biomass fly ash. Setting time, compressive strength, water absorption and bulk density tests were performed on fresh and hardened mortar. While all activation methods influenced the compressive strength development of cement mortar with fly ash, sieving of the biomass fly ash enhanced the early compressive strength of cement mortar. The use of active silica in the form of silica fume ensured higher compressive strength results than those of control specimens throughout the entire measurement period.

## 1. Introduction

The consumption of cement has been soaring in the first decades of the 21st century. Cement production is a source of 7–8% of global carbon dioxide emissions, mainly through the decomposition of carbonate minerals during clinker production [1]. Thus, the overreliance of all modern economies on cement binders is a significant concern for meeting carbon neutrality targets in the decades ahead. This challenge is recognized not only by science, but public discourse as well [2].

One of the key means by which to reduce cement consumption in the construction sector is the usage of mineral additives or the utilization of composite cements in concrete production. One of the most important mineral additives in concrete and a secondary constituent of cement is fly ash (on par with ground-granulated blast-furnace slag), especially siliceous fly ash [3,4]. The global production of coal fly ash is estimated to be at least 750 million tons per annum. Japan is the global leader in the utilization of coal combustion by-products, with a utilization rate above 90%. The global average utilization rate of coal fly ash is about 25%, but in European Union countries and in the United States it is significantly higher and has been growing for years [5,6]. For instance, in 2018 only 1.6% of coal fly ash produced in Poland was disposed [7]. Moreover, the share of coal in overall electricity generation will be systematically reduced in the coming decades across the globe. According to government documents (Polish Energy Policy to 2040), Poland will reduce its coal energy production to 60% of current levels by the end of this decade [5]. Even now, good-quality fly ash has become a valuable resource. The present scarcity of it is a growing issue for the concrete industry, especially in times of increasing economical and legal pressure on cement producers to provide more ecological products.

Similarly, the energy industry has been experiencing structural changes that derive from mounting pressure on it to move to more environmentally friendly sources of energy. One of the strategic routes taken by the European and Polish energy industries to tackle this issue is investment in biomass combustion. The European Environment Agency has revealed that the use of biomass in large combustion plants in the European Union has grown three times between 2004 and 2016 [8]. In 2017 almost 11% of energy produced in Poland came from renewed sources of energy, mainly from firing woody biomass [9].

The management of biomass fly ash, as a novel industrial waste, is a challenge for the Polish energy sector [8]. Whereas in various countries of the European Union adequate legal frameworks help utilize it in the forestry industry or the mining sector, Polish law prohibits this kind of practice due to concerns around heavy metal leaching. In Nordic countries there is a long history of using biomass fly ash as a fertilizer or soil improver in forestry and agriculture. In both Finland and Denmark there is national legislation regulating ash fertilizers [10,11,12]. Utilization of this fly ash in concrete production could provide a sustainable route for its utilization, due to the substantial ability of cement matrix to immobilize heavy metals.

The EN 450-1 standard defines fly ash as a fine powder, consisting of mainly spherical particles composed of alumino-silicate glass. It is a by-product of coal firing or co-firing that is collected from fume gas by electrostatic precipitators. The EN 450-1 standard permits the usage of fly ash derived from the co-combustion of coal with various biomass types, e.g., wood and woody materials, in concrete production. The maximum content of biomass material in the fuel can reach up to 50% for the co-firing of wood. The maximum content of co-fired fly ash in the binder is 30% of its weight. Co-fired fly ash has to meet similar physical and chemical requirements as coal fly ash, e.g., the sum of SiO_2_, Al_2_O_3_ and Fe_2_O_3_, finesses or strength activity index [13,14].

The properties of biomass fly ash can vary and are dependent on various characteristics of the combustion process, e.g., fuel composition, temperature or technology [15,16]. Biomass fly ash that derives from the combustion of herbaceous energy crops tends to have a higher percentage of alkaline oxides in its chemical oxide composition than coal fly ash, whereas woody biomass fly ash tends to have a higher content of calcium oxide (up to 80%). Even so, SiO_2_ content can be as high as 68%, and the overall chemical oxide composition of this type of fly ash can be similar to that of Class C fly ash [17,18,19,20]. Mixed reports have been published on the mineralogical composition and content of reactive phases in biomass fly ashes [21,22,23,24]. The most important minerals found in this type of fly ash are lime, quartz, sylvite, arcanite and anhydrite. It also can contain portlandite, amorphous phases or gypsum [17]. Biomass fly ash particles have a mainly irregular shape and are bigger (d_50_ about 110–150 µm) in size than coal fly ash [25,26]. The combustion temperature drastically influences the amount of unfired carbonate residue in fly ash and the amount of amorphous minerals. A temperature of combustion above 1000 °C drastically decreases the amount of carbonates and elevates the amount of lime in the chemical composition [17]. Elevated levels of chlorines and sulfates were detected in some Polish biomass fly ash [10].

The replacement of 20% of the binder mass of cement with woody biomass fly ash can result in similar values of the hydration peak to pure cement binder specimens [27]. However, a 20% replacement rate can delay the initial and final setting time of cement paste [26]. The mechanical properties of cement composites can be enhanced with wood waste fly ash by a replacement rate of up to about 10% [27]. A substantial increase in 7-day compressive strength was observed with the replacement of 25% of the cement with wood waste fly ash [28]. There are mixed reports on the pozzolanic activity of biomass fly ash [22,27]. Some contribution of pozzolanic reactions to the enhancement of compressive strength was observed in some studies [29,30,31]. However, no additional compressive strength increase after 90 days of curing was observed in others [32,33].

To enhance its properties, fly ash can be activated via various physical, mechanical or chemical methods. These methods might involve altering its oxide or mineralogical composition. Mechanical activation by grinding and physical activation by sieving have been proven to be effective in improving high-calcium coal fly ash utility in the production of cement composites [34,35]. Chemical activation with Na_2_CO_3_ or Al_2_(SO_4_)_3_ of this kind of fly ash can enhance the compressive strength results of cement mortars [36,37]. Reactive silicate materials (e.g., silica fume) could be used as an addition to high-calcium fly ash to modify its alumino-silicate composition and enhance its pozzolanic reactivity [34]. Considering the chemical and mineralogical composition of biomass fly ash, which often lacks amorphous components and is rich in calcium compounds, some of the aforementioned activation methods might be promising in improving the utilization of biomass fly ash in the concrete industry.

Previous works on this subject by the authors were centered around assessing the influence of locally acquired biomass fly ash on cement composites [38], comparing it with the influence of siliceous fly ash [39] and establishing the possibility of enhancing the properties of cement composites modified with biomass fly ash by utilizing asphalt emulsion [40].

The aim of this study is to assess the influence of mechanical activation and activation through the addition of silica fume to fly ash on selected properties of cement composites. The setting time of a blended cement binder, compressive strength, water absorption and hardened density results of blended cement mortar were measured.

## 2. Materials and Methods

A commercial CEM I 42.5R cement was used as a binder material. The material conformed to the requirements of the EN 197-1 standard [41]. Biomass fly ash (BFA) was acquired from the local combined heat and power plant and was produced by the fluidized bed combustion (750 °C) of woody material. Silica fume (SF) that conformed to the requirements of the EN 13263-1+A1 standard [42] was used. Chemical oxide compositions of the fly ash and silica fume are presented in Table 1. Main oxides in fly ash compositions were calcium oxide, potassium oxide and silica oxide. Silica fume is a highly pure form of active amorphous silica.

Selected physical properties of the fly ash are presented in Table 2, and selected physical properties of the silica fume are presented in Table 3. Both strength activity indices (SAIs) of the fly ash prior to its activation were below the EN 450-1 requirements of, respectively, 75% and 85% [14].

The grain-size distribution of fly ash is presented in Table 4. The amount of particles with a diameter above 45 µm was 38.2%. Standard sand, certified in accordance with EN 196-1, was used [43]. Tap water was used for mixing and curing.

Three types of biomass fly ash were used: mechanically activated by grinding (FA(B)G), physically activated by sieving (FA(B)S) or untreated fly ash (FA(B)N) to compare their influence on the properties of cement composites. Sieved fly ash was sieved with a 125 µm sieve. Ground fly ash was ground for 4 h in a laboratory ball mill until most of particles passed through the 125 µm sieve. The aforementioned activation methods were compared with activation by the silica fume replacement: 0%, 20% or 40% of activated fly ash mass (sf/(sf + fa)) was substituted with a more active pozzolanic material, silica fume. Based on those assumptions the mix proportions of cement mortars were prepared and presented in Table 5.

For each mortar series twelve 40 × 40 × 160 mm mortar specimens were casted following EN 196-1 guidance [43]. For each series compressive strength tests were performed after 7, 28 and 90 days of curing. Water absorption and density test were performed after 28 days of water curing. Each compressive strength test was performed on three specimens in accordance with EN 196-1 [43]. Three specimens were used for water absorption and density tests following PN-B-04500 standard instructions [44].

Setting time tests were performed on cement paste specimens prepared in accordance with the EN 196-3 standard [45]. The proportions of the binder corresponded to proportions of cement mortars. The amount of water in the cement paste mix was based on the normal consistency test described in EN 196-3. Cement paste mixes with the water content necessary to achieve their normal consistency are presented in Table 6.

## 3. Results

### 3.1. Characteristics of Activated Fly Ash

**Figure 1:**
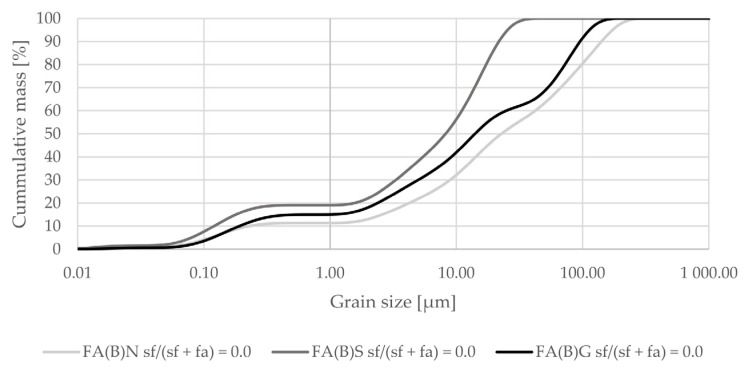
Grain-size distribution of fly ash activated by sieving or grinding compared to the grain-size distribution of untreated fly ash (logarithmic scale).

Mechanical and physical activation changed the grain-size distribution of fly ash (Figure 1). Sieving decreased the maximum grain size of fly ash and shifted its distribution towards smaller particle sizes. Median size was around 20 µm. Grinding did not change the grain-size distribution curve up to around 10 µm, and resulted in a substantially smaller amount of particles at sizes around 60 µm.

**Figure 2:**
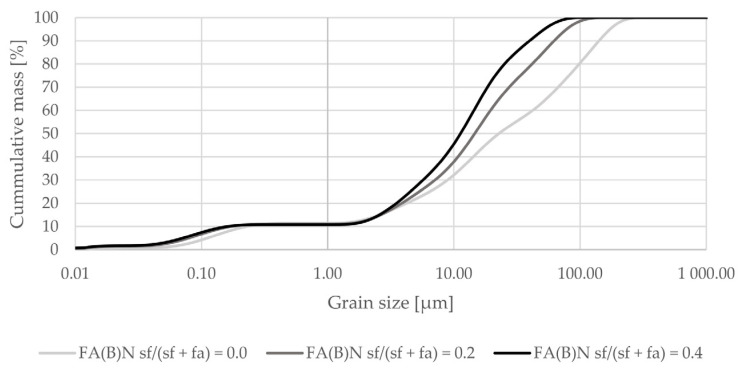
Grain-size distribution of fly ash activated by 20% or 40% of silica fume compared to the grain-size distribution of untreated fly ash (logarithmic scale).

**Figure 3:**
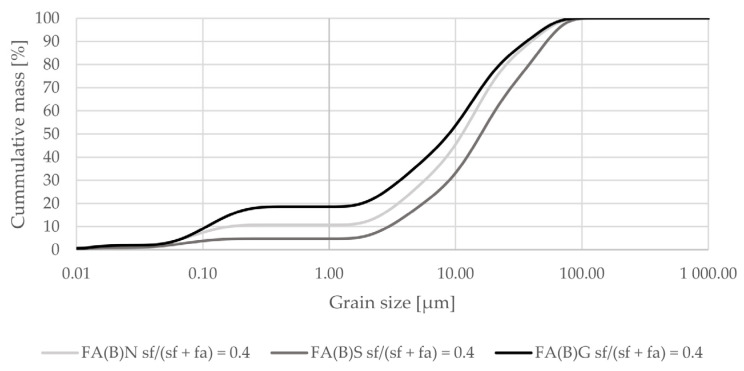
Grain-size distribution of fly ash activated by the addition of 40% of silica fume compared to the grain-size distribution of untreated fly ash (logarithmic scale).

Activation by silica fume addition in fly ash decreased the amount of particles at sizes between 0.2 and 5 µm in the overall distribution (Figure 2). When added as a 40% additive to fly ash it increased the amount of particles in the range of 25–75 µm (Figure 3).

Different means of modification resulted in different water demands of activated fly ash, expressed as the amount of water needed to achieve a normal consistency (Table 6). Sieved fly ash needed 11% more water than untreated fly ash while ground fly ash required 6% more than untreated fly ash. The addition of 20% of silica fume to fly ash resulted in a marginal difference in water demand when ground or sieved fly ash was used. It might imply a good interaction of both activation means on the modification of fly ash compactness and its specific surface. Sieved biomass fly ash had a higher 28-day strength activity index, which was also higher than the EN 450-1 requirement of 75% (Table 7).

**Figure 4:**
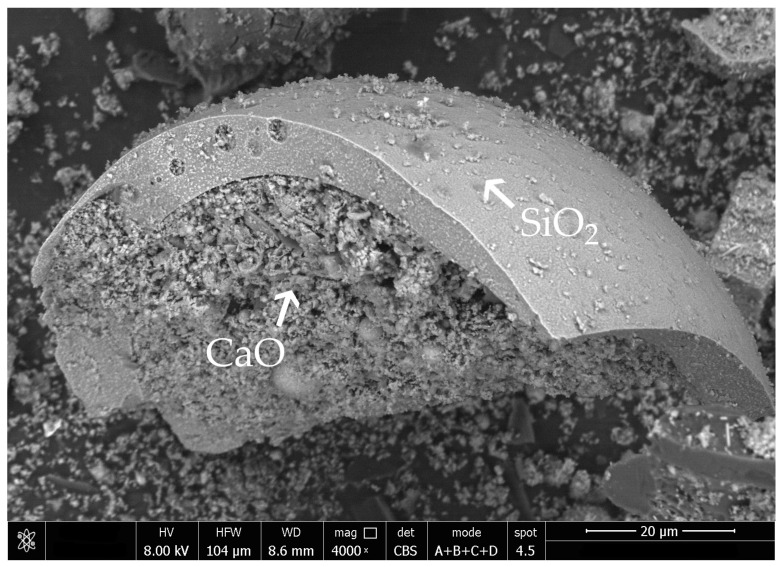
SEM photography of ground biomass fly ash with visible calcium oxide particles inside a broken silicate sphere.

Untreated fly ash had a considerable amount of calcium oxide in its chemical oxide composition (Table 8). Both the sieving and grinding of fly ash resulted in an increase in calcium oxide detected through XRF testing. Silica fume addition provided a substantial amount of active amorphous silicon oxide in the composition, which is needed for pozzolanic reactions (Table 9). As a result, the amount of silica in specimens with 20% and 40% of silica fume in activated fly ash was above the 25% requirement of the EN 450-1 standard. Grinding exposed an additional amount of calcium oxide that was visible during SEM-EDS (Scanning Electron Microscopy-Energy-dispersive X-ray Spectroscopy) analysis, which was available for chemical reactions of the hydration process (Figure 4).

### 3.2. Setting Time Results

**Figure 5:**
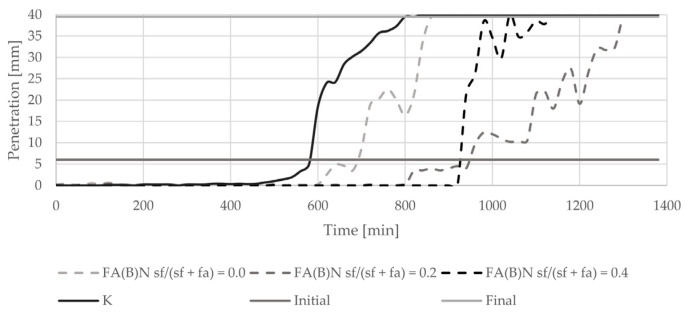
Setting time results of cement pastes with biomass fly ash activated by silica fume addition.

**Figure 6:**
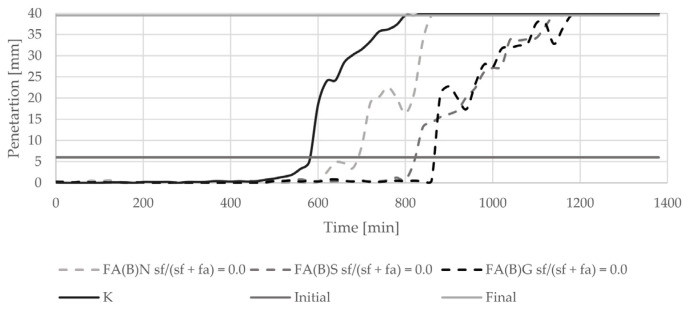
Setting time results of cement pastes with biomass fly ash activated by sieving or grinding.

The introduction of biomass fly ash to cement paste delayed the initial setting time by about 100 min (Figure 5). With an increasing amount of silica fume in the binder, the final setting time of cement paste was delayed. The delay in final setting time was highest with the addition of 20% of silica fume to fly ash. The setting of cement paste with the addition of 40% of silica fume was abrupt. Sieving and grinding clearly influenced the setting time results of specimens with activated fly ash (Figure 6). Both methods delayed the setting of cement paste in a similar manner.

### 3.3. Compressive Strength Results

**Figure 7:**
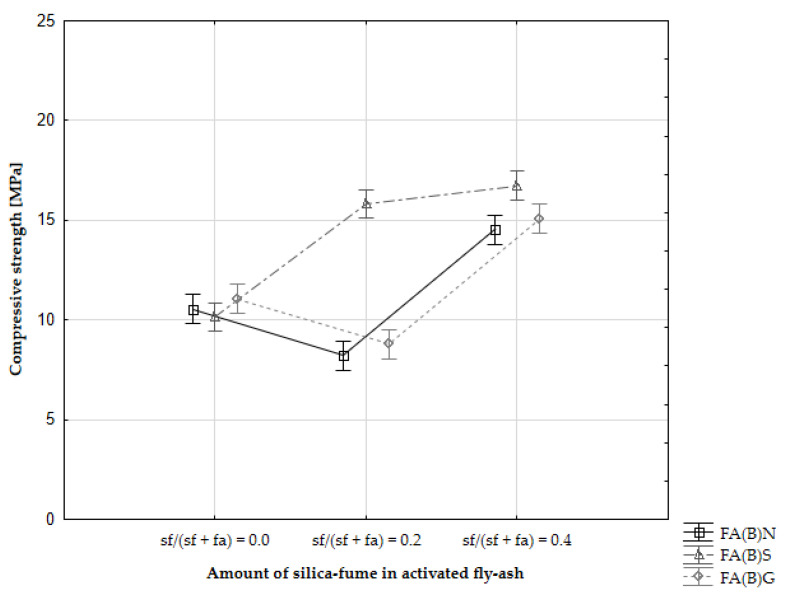
Two-day compressive strength results of cement mortars with activated biomass fly ash (ANOVA certainty in whiskers).

The early compressive strength results showed an interaction between sieving and silica fume addition in activated fly ash. Specimens with sieved fly ash activated by the addition of silica fume had substantially higher 2-day compressive strength results than with ground or untreated fly ash (Figure 7). According to particle-size distribution analysis, sieved fly ash has a similar potential to densify the cement matrix as ground fly ash when silica fume was in the composition (Figure 3). Sieving slightly increased the amount of calcium oxide in biomass fly ash, which could affect the binding process through its hydraulic properties.

**Figure 8:**
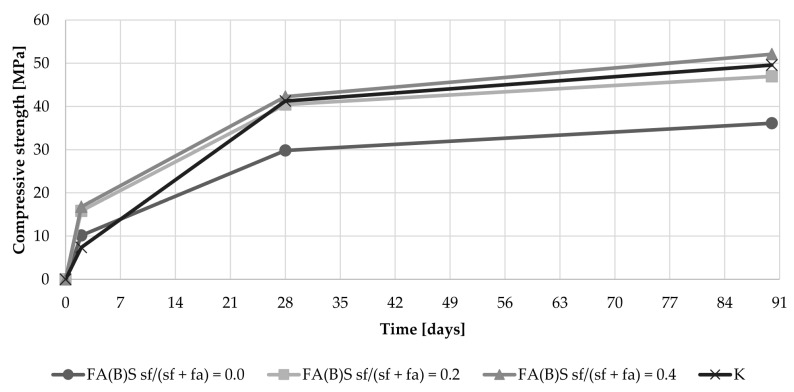
Compressive strength development of cement mortar with the addition of fly ash activated by sieving.

The addition of 20% of silica fume to the fly ash composition resulted in compressive strength development similar to that shown by the control specimens (Figure 8). A substantial increase in 2-day compressive strength was observed especially with cement mortars, which were modified with sieved fly ash and silica fume simultaneously. Specimens with untreated fly ash and without silica fume in the mix had early compressive strength results around 11 MPa. Specimens with the addition of 20% of silica fume to the untreated fly ash mix had results around 8 MPa, while the 2-day compressive strength of sieved fly ash enriched with the addition of 20% of silica fume increased to 16 MPa. The results were on par with specimens with the addition of 40% of silica fume, with two-way ANOVA analysis showing no significant difference between those sets of results.

The effect of biomass activation though sieving on the development of the late compressive strength of specimens was marginal. However, the activation of the fly ash through the addition of 40% of silica fume caused a slightly more dynamic increase in compressive strength between the 28^th^ and 90^th^ day (Figure 8).

### 3.4. Water Absorbtion and Bulk Density Results

**Figure 9:**
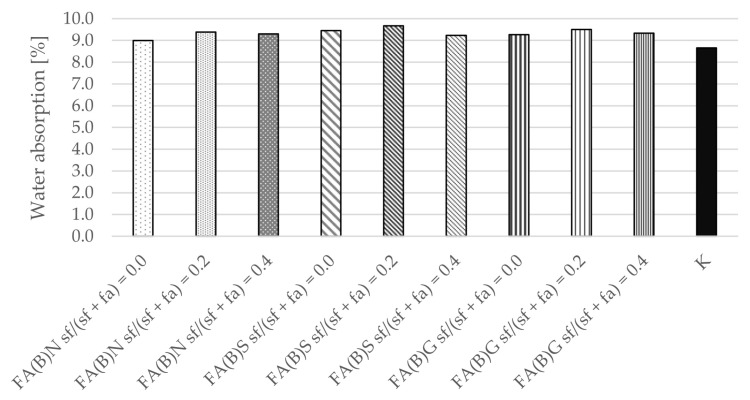
Water absorption of cement mortar with addition of activated and untreated fly ash.

Water absorption tests were performed after 28 days of curing. The differences between the calculated mean results were small between series with the addition of either activated or untreated fly ash (Figure 9). The results of control specimens were slightly smaller than those with fly ash. The series with the addition of 20% of silica fume were characterized by marginally higher water absorption results, while those with the addition of 40% of silica fume in fly ash were comparable to the control specimens.

**Figure 10:**
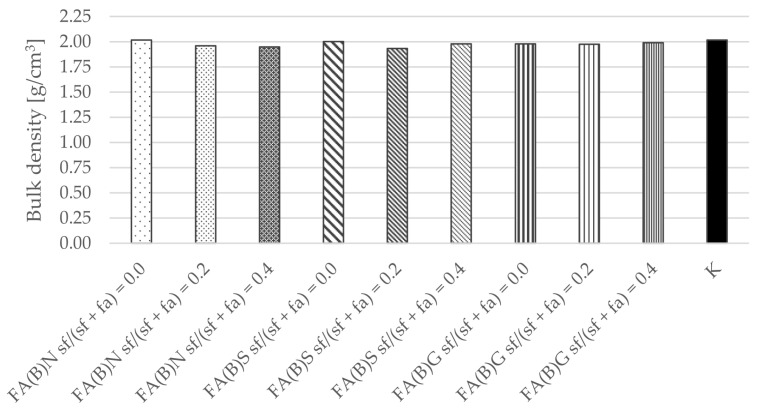
Bulk density of cement mortar with the addition of activated and untreated fly ash.

Similarly, the calculated results of density were similar between series, which indicate that despite the differences in water demand between different fly ash compositions and their activation method, the workability of specimens were not affected and remained comparable (Figure 10).

## 4. Discussion

The results of grain-size distribution analysis indicate that untreated biomass fly ash might need additional modification before utilization in cement composites. Before activation, the maximum particle size of biomass fly ash can be over 250 µm. Both sieved and ground fly ash have a very compacted distribution of particles, which enable them to leave less empty space in the cement matrix. This potentially can lead to better mechanical properties.

The results of the setting time test are in line with the nature of the induction period presented in the literature [4]. The biomass fly ash in the binder is a source of additional calcium ions, the high concentration of which, during early stages of hydration, prevents cement minerals from reacting. Due to the higher content of calcium oxide in fly ash activated with 20% of silica fume than in fly ash activated with 40% of silica fume, the retardation of the setting process is longer. The setting of cement paste with the addition of 40% of silica fume was abrupt—the difference between the initial and final setting time was only 40 min. The changes in the binding timing are in line with other observations conducted and discussed by Kurdowski and Nocuń. The presence of reactive silica in the early hydration stage can be responsible for the bonding of calcium ions with a C-S-H formation, and thus can be responsible for the quickening of the hydration process [4].

The rapid change in the early compressive strength results seen in all specimens with sieved fly ash might indicate the effect of better packing [46]. The shift in the particle-size distribution towards smaller particle sizes might be a factor that caused the initial enhancement of compressive strength. Biomass fly ash can exhibit self-hardening properties due to the high amount of calcium oxide in its composition. Moreover, sieving elevated the amount of calcium oxide in the fly ash. These self-hardening properties can have a direct influence on the early compressive strength results and be responsible for additional strength gain in the first two days of curing. Based on SEM observations, it was established that calcium oxide is present on the surface of silica in the fly ash and that the shift in the particle-size distribution of sieved fly ash towards a higher number of smaller particles can also enhance the availability of calcium oxide during the hydration process, as well as increase the hydraulic reactivity of that material in the results [47,48].

The bulk density results of hardened cement mortar were not affected by the activation of fly ash. Therefore, it can be assumed that the difference between the water demand of fly ash did not affect the results of the study. The differences in the results between the specimens of series thus have to be explained by the difference in the quality of the cement matrices that resulted from the utilization of fly ash.

Water absorption results were only slightly influenced by the activation of biomass fly ash. The results of specimens consisting of 20% of silica fume in activated fly ash were in fact slightly higher than in those with untreated fly ash. The capillary absorption of water is influenced by the compactness of the cement matrix and resulting from its continuity of capillary pores. Therefore, the effect of an increase in compressive strength results should go in line with a decrease in water absorption results. The opposite was observed with the results of sieved fly ash specimens modified with 20% of silica fume.

It can be assumed that not only physical influences in the form of the modification of the particle-size distribution, but chemical interactions in the form of hydraulic and pozzolanic reactions between the compounds of fly ash and silica fume were also responsible for the enhancement of compressive strength results.

## 5. Conclusions

The sieving and grinding of biomass fly ash changed its particle-size distribution. Both activation methods created a mixture with a higher number of small particles, which enabled a better packing effect of the additive in the cement matrix to be achieved. The water demand of sieved biomass fly ash was higher than that of ground biomass fly ash—sieved fly ash raised it by 11% and ground fly ash by 6%.

The addition of silica fume to fly ash had a dominant influence on the results observed during this study. The addition of silica fume resulted in higher 2-day compressive strength results than those of the control specimens. The results of untreated fly ash enriched with 40% of silica fume (16.4 MPa) were more than 90% higher than the results of the control specimens (7.4 MPa). Specimens with the addition of silica fume to fly ash had comparable 28-day and 90-day compressive strength results to control specimens. The highest results can be associated with the high pozzolanic reactivity of the additive, potential interaction with calcium in fly ash and the particle-size distribution of the fly ash activated by this method.

Sieving and grinding had a substantial influence on the 2-day compressive strength results. Sieving of the fly ash resulted in a 37% increase in compressive strength results compared to the compressive strength of the control specimen, which lead to an increase in compressive strength from 7.4 MPa to 10.2 MPa. Both the sieving and grinding of the biomass fly ash interacted with the addition of silica fume and increased further the early compressive strength results when utilized simultaneously in the series. This was especially observable with specimens activated by the addition of 20% of silica fume in fly ash mass, where the results were two times higher than those of the control specimens. After additional curing time, the influence of sieving or grinding was marginal for the 90-day compressive strength results. The early compressive strength results suggest some form of chemical interaction between the fly ash and reactive silica that had an immediate effect on the quality of the cement matrix.

The water absorption and bulk density results of hardened mortar were comparable between the specimens of different series. The water absorption results of the specimens were in the range of 9–9.5%. The water absorption results were slightly (0.5 percentage points) higher with the addition of 20% of silica fume to the fly ash.

The results presented in this study encourage additional research on the activation of biomass fly ash with a goal of obtaining a valuable additive for the production of environmentally friendly concrete. Present findings show that both the grinding and sieving of fly ash paired with mineralogical activation through the addition of active silica can enhance the amount of fly ash that can be utilized in cement composites without negative effects on key properties, such as compressive strength and water absorption. Additional studies must be conducted to find the optimal range in which sieved and ground biomass fly ash can be added to concrete with active silica in the additive composition to fully utilize the physical and hydraulic properties of biomass fly ash.

## Figures and Tables

**Table 1:** Chemical Oxide Composition of Biomass Fly Ash and Silica Fume.

Oxide	Silica Fume	Biomass Fly Ash
SiO_2_	90.9	9.6
Al_2_O_3_	0.0	2.4
P_2_O_5_	0.2	0.0
K_2_O	2.6	14.7
CaO	0.8	56.1
MnO	0.6	3.1
Fe_2_O_3_	4.5	7.9
CuO	0.1	0.0
ZnO	0.2	0.5
As_2_O_3_	0.1	0.0
SO_3_	0.0	4.3
TiO_2_	0.0	0.6
Cl	0.0	0.7
BaO	0.0	0.1

**Table 2:** Physical Properties of Biomass Fly ash.

Property	Biomass Fly Ash	EN 450-1 Requirements
Fineness	66.4%	≤40% (N category)
Loss on ignition	8.0%	≤9% (C category)
Strength activity index after 28 days	65.1%	≥75%
Strength activity index after 90 days	66.9%	≥85%

**Table 3:** Physical Properties of Silica Fume.

Property	Silica Fume
Specific surface	15–35 m^2^/g
Loss on ignition	<4.0%
Bulk density	<350 kg/m^3^

**Table 4:** Grain-Size Distribution of Biomass Fly Ash (%).

Material	>2.0 mm	2.0–1.0 mm	1.0–0.5 mm	0.50–0.25 mm	0.250–0.125 mm	0.125–0.063 mm	0.063–0.045 mm	0.045–0.000 mm
Biomass fly ash	0.0	0.0	0.0	0.3	12.0	18.7	7.2	61.8

**Table 5:** Cement Mortar Mix Proportions (g).

Series Code	Silica Fume	Biomass Fly Ash			Cement	Sand	Water
		Untreated	Sieved	Ground			
FA(B)N sf/(sf + fa) = 0.0	0.0	112.5	0.0	0.0	337.5	1350	225
FA(B)N sf/(sf + fa) = 0.2	22.5	90.0	0.0	0.0	337.5	1350	225
FA(B)N sf/(sf + fa) = 0.4	45.0	67.5	0.0	0.0	337.5	1350	225
FA(B)S sf/(sf + fa) = 0.0	0.0	0.0	112.5	0.0	337.5	1350	225
FA(B)S sf/(sf + fa) = 0.2	22.5	0.0	90.0	0.0	337.5	1350	225
FA(B)S sf/(sf + fa) = 0.4	45.0	0.0	67.5	0.0	337.5	1350	225
FA(B)G sf/(sf + fa) = 0.0	0.0	0.0	0.0	112.5	337.5	1350	225
FA(B)G sf/(sf + fa) = 0.2	22.5	0.0	0.0	90.0	337.5	1350	225
FA(B)G sf/(sf + fa) = 0.4	45.0	0.0	0.0	67.5	337.5	1350	225
K	0.0	0.0	0.0	0.0	450.0	1350	225

**Table 6:** Cement Paste Mix Proportions (g).

Series Code	Silica Fume	Biomass Fly Ash			Cement	Water
		Untreated	Sieved	Ground		
FA(B)N sf/(sf + fa) = 0.0	0	125	0	0	375	160.0
FA(B)N sf/(sf + fa) = 0.2	25	100	0	0	375	175.0
FA(B)N sf/(sf + fa) = 0.4	50	75	0	0	375	185.0
FA(B)S sf/(sf + fa) = 0.0	0	0	125	0	375	178.0
FA(B)S sf/(sf + fa) = 0.2	25	0	100	0	375	180.0
FA(B)S sf/(sf + fa) = 0.4	50	0	75	0	375	190.0
FA(B)G sf/(sf + fa) = 0.0	0	0	0	125	375	170.0
FA(B)G sf/(sf + fa) = 0.2	25	0	0	100	375	171.5
FA(B)G sf/(sf + fa) = 0.4	50	0	0	75	375	183.0
K	0	0	0	0	500	165.0

**Table 7:** Physical Properties of Activated Biomass Fly Ash.

Property	Ground Biomass Fly Ash	Sieved Biomass Fly Ash
Loss on ignition	8.0%	7.8%
Strength activity index after 28 days	70.0%	78.4%
Strength activity index after 90 days	68.3%	70.8%

**Table 8:** Chemical Oxide Composition of Sieved and Ground Biomass Fly Ash Compared with untreated Fly Ash.

Oxide	FA(B)N sf/(sf + fa) = 0.0	FA(B)S sf/(sf + fa) = 0.0	FA(B)G sf/(sf + fa) = 0.0
SiO_2_	9.6	5.9	14.2
Al_2_O_3_	2.4	0.0	0.0
P_2_O_5_	0.0	0.0	0.0
K_2_O	14.7	14.7	14.1
CaO	56.1	61.7	55.4
MnO	3.1	3.4	2.8
Fe_2_O_3_	7.9	8.5	8.1
CuO	0.0	0.0	0.0
ZnO	0.0	0.1	0.1
As_2_O_3_	0.5	0.6	0.5
SO_3_	0.0	0.0	0.0
TiO_2_	4.3	4.2	3.9
Cl	0.6	0.6	0.6
BaO	0.7	0.0	0.0

**Table 9:** Chemical Oxide Composition of Biomass Fly Ash Activated by Silica Fume Addition.

Oxide	FA(B)N sf/(sf + fa) = 0.2	FA(B)N sf/(sf + fa) = 0.4	FA(B)S sf/(sf + fa) = 0.2	FA(B)S sf/(sf + fa) = 0.4	FA(B)G sf/(sf + fa) = 0.2	FA(B)G sf/(sf + fa) = 0.4
SiO_2_	25.9	42.1	22.9	39.9	29.5	44.9
Al_2_O_3_	1.9	1.4	0.0	0.0	0.0	0.0
P_2_O_5_	0.0	0.1	0.0	0.1	0.0	0.1
K_2_O	12.3	9.9	12.3	9.9	11.8	9.5
CaO	45.0	34.0	49.5	37.3	44.5	33.6
MnO	2.6	2.1	2.8	2.3	2.4	1.9
Fe_2_O_3_	7.2	6.5	7.7	6.9	7.4	6.7
CuO	0.0	0.0	0.0	0.0	0.0	0.0
ZnO	0.4	0.4	0.1	0.1	0.1	0.1
As_2_O_3_	0.0	0.0	0.5	0.4	0.4	0.4
SO_3_	3.4	2.6	0.0	0.0	0.0	0.0
TiO_2_	0.5	0.4	3.4	2.5	3.1	2.3
Cl	0.6	0.4	0.5	0.4	0.5	0.4
BaO	0.1	0.1	0.0	0.0	0.0	0.0

## Data Availability

Not applicable.
